# Testing the Coulomb/Accessible Surface Area solvent model for protein stability, ligand binding, and protein design

**DOI:** 10.1186/1471-2105-9-148

**Published:** 2008-03-13

**Authors:** Marcel Schmidt am Busch, Anne Lopes, Najette Amara, Christine Bathelt, Thomas Simonson

**Affiliations:** 1 Laboratoire de Biochimie (UMR CNRS 7654), Department of Biology, Ecole Polytechnique, 91128, Palaiseau, France

## Abstract

**Background:**

Protein structure prediction and computational protein design require efficient yet sufficiently accurate descriptions of aqueous solvent. We continue to evaluate the performance of the Coulomb/Accessible Surface Area (CASA) implicit solvent model, in combination with the Charmm19 molecular mechanics force field. We test a set of model parameters optimized earlier, and we also carry out a new optimization in this work, using as a target a set of experimental stability changes for single point mutations of various proteins and peptides. The optimization procedure is general, and could be used with other force fields. The computation of stability changes requires a model for the unfolded state of the protein. In our approach, this state is represented by tripeptide structures of the sequence Ala-X-Ala for each amino acid type X. We followed an iterative optimization scheme which, at each cycle, optimizes the solvation parameters and a set of tripeptide structures for the unfolded state. This protocol uses a set of 140 experimental stability mutations and a large set of tripeptide conformations to find the best tripeptide structures and solvation parameters.

**Results:**

Using the optimized parameters, we obtain a mean unsigned error of 2.28 kcal/mol for the stability mutations. The performance of the CASA model is assessed by two further applications: (i) calculation of protein-ligand binding affinities and (ii) computational protein design. For these two applications, the previous parameters and the ones optimized here give a similar performance. For ligand binding, we obtain reasonable agreement with a set of 55 experimental mutation data, with a mean unsigned error of 1.76 kcal/mol with the new parameters and 1.47 kcal/mol with the earlier ones. We show that the optimized CASA model is not inferior to the Generalized Born/Surface Area (GB/SA) model for the prediction of these binding affinities. Likewise, the new parameters perform well for the design of 8 SH3 domain proteins where an average of 32.8% sequence identity relative to the native sequences was achieved. Further, it was shown that the computed sequences have the character of naturally-occuring homologues of the native sequences.

**Conclusion:**

Overall, the two CASA variants explored here perform very well for a wide variety of applications. Both variants provide an efficient solvent treatment for the computational engineering of ligands and proteins.

## Background

Solvation effects play an important role in protein folding and stability. Likewise, the processes of protein:protein and protein:ligand binding are accompanied by effects such as desolvation and rearrangement of solvent molecules. These solvation effects can be calculated by explicit solvent models, such as molecular dynamics simulations using a sphere of water molecules [1]. For certain large-scale applications, however, this explicit solvent treatment is too time-consuming. In protein design, an enormous number of amino acid sequences need to be considered [2]. Likewise, the screening of a large library of ligand molecules as a function of their binding affinity to a protein is a costly procedure and requires efficient methods. To avoid these problems, implicit solvent models are often used, which yield significant computational efficiency [3]. They do not consider the solvent degrees of freedom explicitly, but treat the solvent as a continuous medium having the average properties of the real solvent. Empirical methods, such as the solvent accessible surface area (ASA) model [4], often provide simple and quick ways of evaluating the solvation energy with an accuracy comparable to theoretical models. ASA models have become widely accepted within available implicit solvent treatments and have been used successfully in many applications, such as protein molecular dynamics [5-7], structure prediction [8] and protein:ligand binding [9,10]. In the ASA approach, the solvation free energy of a solute is expressed as a sum of atomic contributions, weighted by their solvent-exposed area. The contribution of each atom is quantified by a surface coefficient, which reflects the hydrophobicity or hydrophilicity of the particular atom type.

Apart from non-polar contributions to the solvation free energy, such as the entropy cost for cavity formation and van der Waals interactions, electrostatic contributions play an important role. Due to the fitting of the ASA model to experimental data, the electrostatic contribution is partly incorporated into the parameters. However, especially when using a small number of atom types, it is necessary to additionally calculate a screening energy, which accounts for the shielding of protein-protein electrostatic interactions by the high dielectric solvent. A simple approach is to add a term that reduces the electrostatic interactions between protein atoms by a constant factor, *ε*, which plays the role of a dielectric constant. To balance the two components of the solvent model, an overall weight *α *is applied to the ASA term. This combined model is known as the Coulomb/Accessible Surface Area (CASA) model [3]. More accurate approaches for screening energy calculations are the Generalized Born (GB) model [11,12] and the Poisson-Boltzmann (PB) model [13-15]. A disadvantage of these methods, however, is that they are not easily pairwise decomposable [16]: the energy is not expressed as a sum over atom pairs. Further, they are relatively time-consuming compared with surface area based models.

The first set of atomic solvation parameters distinguishing between different atom types was developed by Eisenberg and McClachlan in 1986 [4]. They used octanol to water transfer energies for 20 amino acids to derive solvation coefficients for 5 atom types. Subsequently, a number of studies have been devoted to the parameterization of the atomic surface coefficients. They differ in the assignment of atoms to characteristic groups and in the experimental data that were used to fit the coefficients. Ooi et al. [17] used 7 different atomic coefficients, fitted to experimental free energies of solvation of small organic molecules. Fraternali and van Gunsteren [6] restricted the atom types to only two: one for carbon, representing the hydrophobic effect, and one for both nitrogen and oxygen, representing the hydrophilic effect. These two parameters were optimized, such that the hydrophobic and hydrophilic solvent-accessible surface areas in proteins obtained from MD simulations matched those measured from the corresponding X-ray structures. Later, several more highly-parameterized ASA models [10,18,19] were developed, which use up to 100 different atom types and large training sets of experimental solvation free energies for diverse organic molecules.

In previous work [20], we optimized the CASA model for side chain placement and mutagenesis. We modified the atomic solvation parameters of Fraternali [6] by including an additional surface coefficient for atoms in charged groups, and by optimizing the dielectric constant *ε *and the weight *α *of the surface term. The model was optimized and tested using sidechain reconstruction calculations, protein solvation energies, and stability changes for point mutations involving the insertion or removal of a charged sidechain. In this paper, we continue to explore the performance of the CASA model, pursuing two directions. First, we consider whether a specific treatment of aromatic groups can lead to an improved parameterization. Second, we consider a broader set of test calculations than before. We again consider stability changes, but we include a wider variety of mutations, most of which do not affect charged groups. We consider the calculation of protein:ligand binding free energy changes due to point mutations, a very important application. So far, most ligand binding studies using ASA models used a large set of atomic surface coefficients. Pei et al [10], for example, used 100 atom types and reproduced the binding free energies of a test set of 50 protein:ligand complexes with a standard error of 2.0 kcal/mol. Our own model is much less heavily parameterized, but yields a comparable accuracy.

Finally, we perform automated protein design for eight small proteins of the SH3 family. Computational protein design is another area that requires good implicit solvent models [2,21,22]. This approach can help engineer new proteins as well as predict protein structure. It considers a given backbone structure of a protein and predicts the amino acid sequences that fold into it [23-30]. This information can be used either to identify mutations that stabilize a given structure or to assign the given 3D structure to new sequences with yet undetermined protein structures. The protein design procedure is applied here to a small test set of eight SH3 proteins and the performance of the optimized solvation parameters is compared to that of the earlier parameter set.

The newer CASA parameterization derived here treats aromatic groups as a specific group. All the model parameters are then reoptimized from scratch. Most studies so far have used experimental transfer free energies from octanol or cyclohexane to water for small model molecules to derive solvation parameters. However, organic solvents are only a crude approximation of the protein interior [9]. A more recent study by Zhou et al. [31] used a database of protein mutation experiments to develop atomic solvation parameters. With these parameters, the binding free energies of 21 protein-protein complexes were predicted with an rms deviation of 2.3 kcal/mol. Likewise, Lomize et al. [32] achieved very good agreement with experimental data using atomic solvation parameters based on protein stabilities. Here, we use a similar approach and derive our newer solvation parameters from experimental protein and peptide stability changes. We employ a procedure that attempts to match the computed stability changes to the experimental values, using a set of 140 mutations. Simultaneously, the model for the unfolded reference state of a protein is optimized: for each amino acid type, a preferred unfolded conformation is chosen from a large library of tripeptide fragments obtained from various proteins.

The final parameter set yields improved performance for protein stabilities, as expected. The newer and the earlier parameters yield comparable, good performance for ligand binding and protein design. Given the importance of implicit solvent models in the fields of structure-based drug design, prediction of protein structure and protein design, both of our optimized CASA models should be valuable tools for a wide range of applications.

## Results

### CASA parameter optimization and stability calculations

In recent work, we optimized and tested the CASA model, using just three atomic categories and performing sidechain reconstruction and protein stability calculations. We refer to the corresponding set of atomic surface coefficients as the "modified Fraternali", or MF parameters (Table 1). Indeed, the starting point for the earlier optimization was the parameter set of Fraternali & van Gunsteren [6]. Here, we want to test the CASA model further, and to explore whether a more extensive parameterization will improve its performance. Our previous CASA model [20] included only polar, unpolar and ionic atom coefficients. The current protocol also distinguishes aromatic atoms as a separate group. This refined atom assignment may be expected to improve the solvent model, as it has been shown that aromatic groups have solvation properties different from aliphatic groups [33]. With this modification, we reoptimized all the model parameters from scratch. We scanned a range of values for the four different surface coefficients; additionally, three different values for the dielectric constant *ε *in the Coulomb screening term (Eq. 2) were tested. A data set of 140 experimental stability mutations was used to adjust the parameters: these data correspond to 7 different helical peptides and 3 different proteins. The protein mutations involve mainly ionized residues, while the peptide mutations (which were taken from helix propensity scales) cover the full range of amino acids. In contrast to an earlier study by Lomize et al. [32], which derived atomic solvation parameters based on stability data for the protein interior, and in contrast to our previous study [20], this set of mutations involves mainly solvent-exposed residues (14% buried residues).

**Table 1:** Atomic solvation parameters (kcal/mol/Å^2^) for different atom types

atom type	MF	PHIA
unpolar	0.0119	-0.005
aromatic	0.0119	-0.04
polar	-0.0597	-0.08
ionic	-0.15	-0.10

MF: modified Fraternali parameters; PHIA: parameters optimized here.

To compute the stability of a protein, it is necessary to construct a model for the unfolded state. Here, we assume that sidechains do not interact with each other in the unfolded state, and we describe the environment of each sidechain with a simple tripeptide model. Each sidechain thus interacts only with the local tripeptide backbone and the solvent. For each amino acid type, one preferred structure was chosen from a large set of tripeptide conformations obtained from various proteins.

**Figure 1:**
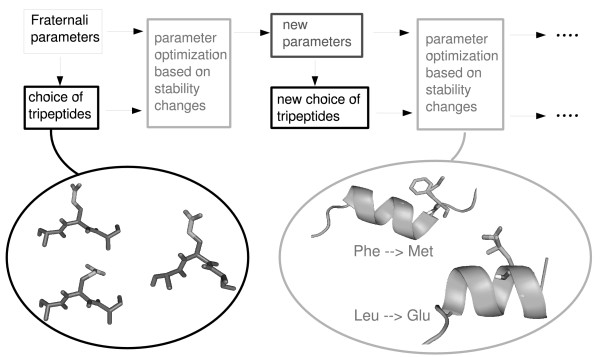
**Iterative parameter optimization**. Iterative optimization of the atomic solvation parameters and the tripeptide models for the unfolded reference state of a protein.

Since the choice of the preferred unfolded reference structure for each amino acid type also depends on the parameterization of the solvent model, the parameter optimization had to proceed iteratively (Figure 1): (i) A preferred tripeptide structure was chosen for each amino acid using the current set of solvation parameters (ii); The solvation parameters were adjusted to best reproduce the experimental stability changes using the current set of reference structures. This was done based on the rms deviation of the computed stability changes from the experimental values.

**Figure 2:**
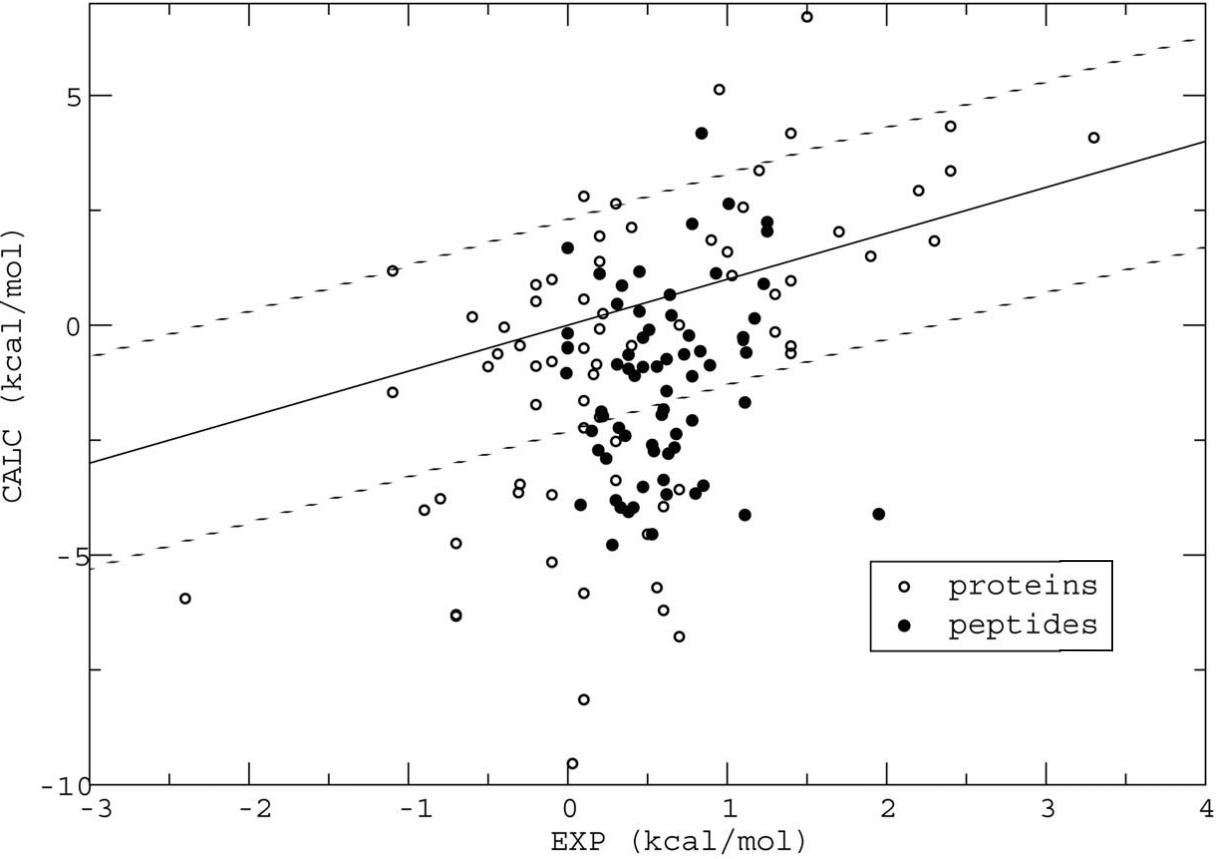
**Stability changes**. Calculated and experimental changes in stability upon mutation for 7 helical peptides and 3 proteins. The solid line corresponds to a (rather poor) linear fit; it and the dashed lines should be viewed as simple guides to help appreciate the error magnitudes.

Starting from the previously optimized solvation parameters [6,20], the optimization procedure converged after 4 iteration cycles. Table 1 shows the set of surface coefficients chosen from the top-ranking results. We refer to the new set as the PHIA set (for "polar, hydrophobic, ionic, aromatic"). Compared with the earlier, MF parameters, the PHIA set shows a clear difference between the aromatic and the unpolar coefficient, a more positive ionic coefficient and a slightly more negative unpolar coefficient. The experimental and computed stability changes are compared in Figure 2. The correlation between the two sets is modest, 43%. Nevertheless, the mean error is reasonable, with an rms deviation of 2.94 kcal/mol for the complete set of experimental values and a mean unsigned error of 2.28 kcal/mol (Table 2). The rank ordering of the data is characterized by a Spearman rank correlation of 29% [34]; the probability of obtaining this value by chance is less than 0.001 (according to Student's test with a *t*-value of 3.5 and 140 degrees of freedom [34]). The PHIA results represent a significant improvement over the performance of the MF parameters, which give an rms deviation of 4.65 kcal/mol and a mean unsigned error of 3.56 kcal/mol. The dielectric constant for the new, PHIA set of surface coefficients was set to 24. A higher dielectric constant of 32 gives only insignificant improvement (rms = 2.93 kcal/mol and mean = 2.27 kcal/mol), while a lower value of 16 leads to noticeably poorer agreement with experiment (rms = 3.23 kcal/mol and mean = 2.52 kcal/mol).

**Table 2:** Rms and mean unsigned error (kcal/mol) for stability mutations

group of data	number of mutations	rms error	mean error
all	140	2.94	2.28
peptides	67	2.68	2.20
proteins	73	3.17	2.34
charged	87	3.06	2.29
uncharged	54	2.72	2.22

In addition to the criterium of minimal deviation from the experimental data, care was taken to select a parameter set that makes sense physically. The ordering of the coefficients should follow the expected preference of solvent exposure of each atom group: unpolar < aromatic < polar < ionized. Further, the coefficients of different groups should be sufficiently separated from each other. The surface coefficient of the unpolar atom group was allowed to be slightly negative, as this led to better agreement with the experimental data. To ensure that this does not lead to unphysical behaviour, the surface coefficient was tested on a propane dimer system. Studies on methane and neopropane association in water report an association free energy of -1.0 and -2.7 kcal/mol, respectively [35,36]. Using values between 0.0119 and -0.01 kcal/mol/Å^2 ^for the nonpolar surface coefficient, the computed association free energy varies from -2.75 to +0.09 kcal/mol. The nonpolar coefficient of -0.005 kcal/mol/Å^2 ^used here gives an acceptable association free energy of -0.56 kcal/mol.

To verify that the parameters were not overly biased by the optimization procedure, we performed cross-validation tests (see Methods). Part of the data (30 mutations) were omitted from the optimization process, then used to test the error level. This led to very similar parameters and errors compared to the original parameter optimization. Specifically, one cross-validation run led to the following optimized parameters (in kcal/mol/Å^2^; the PHIA values obtained above are given in parentheses): aromatic: -0.08 (-0.04); ionic: -0.10 (-0.10); polar: -0.09 (-0.08); nonpolar: -0.005 (-0.005). The optimal dielectric was 24, as before. The mean and rms errors for the omitted data were 2.22 and 3.13 kcal/mol, respectively, compared to 2.11 and 2.70 for the optimization data. The mean and rms errors with the PHIA parameters for the omitted data were similar: 2.32 and 2.92 kcal/mol. The other cross-validation run led to the exact same atomic coefficients and dielectric constant, and to mean and rms errors for the omitted data of 2.08 and 2.68 kcal/mol (compared to 2.16 and 2.71 kcal/mol with the PHIA set). Thus the optimized parameters and the error levels are similar with and without cross-validation, showing that the resulting parameters are fairly robust.

The model performance shows a moderate dependence on the system considered. The peptide models taken alone lead to a slightly better agreement with the experimental data (Table 2), which might be due to their simple helical structure compared with a large protein system (see also Figure 2). The performance of the uncharged mutations is better than that of the charged ones (Table 2). This occurs partly because the proportion of charged mutations is higher for the proteins (mostly charged mutations).

Compared with the results of Lomize et al. [32], which gave an rms deviation from experimental stability changes of only 0.41 kcal/mol, the performance here is less good. In their study, however, the solvation parameters were fitted to a more restricted set of experimental data. Only buried, uncharged residues in *α*-helices and *β*-sheets of proteins were used. In contrast, in our study, both charged and uncharged, solvent-exposed and buried, and protein and peptide mutations were included. Therefore, it can be expected that our more varied set of mutations results in a higher deviation from experiment.

### Binding affinities

The performance of both the earlier, MF and the new, PHIA solvation parameters was assessed on a large set of experimental binding affinities and resistance mutations. These data include 80 mutations in six different ligand-protein systems: (i) tyrosyl-tRNA synthetase in complex with tyrosine (TyrRS), (ii) aspartyl-tRNA synthetase in complex with aspartate (AspRS), (iii) lysozyme in complex with the antibody HyHel-10 (Lyso) and (iv) the complex of the transmembrane glycoprotein CD4 with the gp120 component of the HIV virus (CD4); (v) the BPTI:trypsin complex; (vi) the chymotrypsin:BPTI complex. A seventh system was also studied: the tyrosine kinase Abl in complex with the drug imatinib. For this system, information is only available on mutations leading to resistance to the drug imatinib [37], while for the other four systems, more precise values of the binding affinities are available. The energy function was slightly different from the one used for the stability mutations above. Here, the dielectric constant (Eq. 2) *ε *was set to 16 and the weight *α *of the surface area term was set to 0.5 (instead of *ε *= 24, *α *= 1, above). These values gave improved performance for the binding affinities.

**Figure 3:**
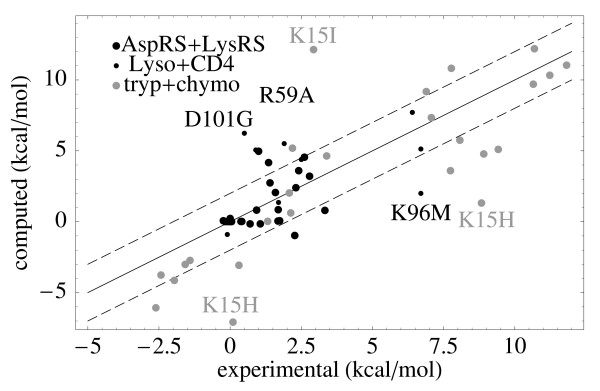
**Binding affinities**. Calculated and experimental differences in  binding affinity upon mutation for 6 different  protein-ligand systems: Aspartyl- and Tyrosyl-tRNA  synthetases (AspRS+TyrRS), a Lysozyme-antibody  complex (Lyso), the CD4 complex with the gp120  component (CD4), and the BPTI complexes with  trypsin and chymotrypsin.

For the first four systems, (i-v; 55 mutations in all), the calculation of binding affinities followed a very simple protocol that only optimizes the conformation of the side chain in the mutated position, while the rotamers of the remaining side chains are left unchanged apart from a slight minimization. Further, the starting structures of the ligand-bound state and the ligand-free state are assumed to be identical except for the side chain at the mutated position. Although this is a rather simple approach, the resulting computed binding affinities are in reasonable agreement with the experimental values. The mean unsigned error for the differences in binding affinities upon mutation is 1.76 kcal/mol with the PHIA parameters, and 1.47 kcal/mol with the MF parameters (Table 3). Thus, the performance of the two CASA variants for this application is similar. The PHIA results for all mutations are given in Additional File 1. The performance is slightly poorer when charged residues are mutated. The more complicated systems such as protein-antibody binding (Lysozyme) or protein-protein binding (CD4) give slightly higher deviations from experiment (see also Figure 3). The mean unsigned errors for the small molecule-ligands (AspRS and TyrRS), combined, is 1.24 kcal/mol with PHIA, while the protein-ligand systems (Lysozyme and CD4), combined, give a mean unsigned error of 2.29 kcal/mol (Table 3).

**Table 3:** Mean error (kcal/mol) for the binding free energies with CASA and GB/SA

data set	number of mutations^*b*^	CASA	GB-ACE	GB-HCT	reference
all^*a*^	55 (52/48)	1.76	2.39	1.96	[66–72]
charged^*a*^	24 (23/20)	2.09	2.70	2.89	
AspRS	9 (9/9)	1.86	3.40	1.25	[73]
TyrRS	15 (15/15)	0.62	0.80	1.13	[66–70]
Lyso	9 (9/7)	2.68	3.01	2.92	[71]
CD4	22 (19/17)	1.89	2.34	2.53	[72]

BPTI^*c*^	25	2.75	-	-	[38, 39]

^*a*^Excluding the BPTI complexes, which were computed with a slightly different protocol and for which GB data are not available. ^*b*^For GB-ACE and GB-HCT, some mutations were excluded due to unfavorable VdW contacts; the numbers of mutations (ACE/HCT) are in parentheses. ^*c*^BPTI:trypsin and :chymotrypsin complexes.

Results were also computed for two other systems, the BPTI:trypsin [38] and BPTI:chymotrypsin [39] complexes. 13 mutations at position 15 in BPTI were studied in each case. The native residue is a lysine. One mutation was excluded (K15W in BPTI:trypsin) due to a large van der Waals contribution to the affinity change (see Methods), leaving 25 mutations. With the simple protocol used above, the agreement with experiment was poorer, with a mean error of 3.4 kcal/mol for the 25 mutations. A slightly different protocol was then tried. The entire BPTI was minimized, instead of just the mutated position (see Methods). This led to improved agreement, with mean errors of 2.68 and 2.81 kcal/mol for BPTI:trypsin and BPTI:chymotrypsin, respectively: about the same level as for the lysozyme:antibody complex. This illustrates the need for more extensive structural relaxation with some mutations. More generally, these two examples show that, not surprisingly, with the simple energy functions and conformational exploration used here, a certain amount of system-specific parameter fitting and adjustment can be necessary.

For the resistance mutations in Abl-kinase, good qualitative agreement was achieved, in so far as all the mutated proteins gave less favorable binding affinities than the native protein (Table 4). For positions 22 and 86, the computed difference between the mutant and native binding free energies may be too small; the other three values, however, are clearly consistent with the observed resistance to imatinib binding.

**Table 4:** Binding free energy differences for the ABL:imatinib complex with CASA and GB/SA

mutation	CASA	GB-ACE	GB-HCT
L17V	1.74	1.00	0.80
Y22F	0.30	0.23	0.10
V58A	6.50	1.23	1.10
F86L	0.46	0.75	2.50
F128V	5.99	5.46	7.10

In kcal/mol.

There are several mutations that are badly predicted, including R59A in the CD4:gp120 system and D101G and K96M in the Lysozyme:antibody system (Figure 3). The first two cases involve a large to small mutation, and all three involve removal of a net charge; these processes which might require a more extensive rearrangement of the surrounding residues, as in the BPTI complexes (where the native residue was a lysine in all 25 mutations). In the simple protocol employed here, the rotamers of the side chains in the vicinity of the mutation are not reoptimized, and the slight minimization carried out here might not be sufficient to model a realistic mutated protein conformation. For the K96M case, we tried the same protocol as for the BPTI cases, applying 50 steps of Powell minimization to the entire lysozyme protein, instead of just the mutated sidechain. This led to a computed binding free energy change of +4.5 kcal/mol, much closer to the experimental value of 7.9 kcal/mol. For one other case, the D78A mutation in TyrRS, we employed a much more extensive conformational search: all the rotamers close to the mutation site were explored using a stochastic search strategy; the error in the binding free energy decreased from 1.9 kcal/mol to 0.8 kcal/mol (not shown). These examples suggest that when the sidechain charge and/or volume changes substantially, a more sophisticated conformational sampling is required. More work in this direction is underway.

On the whole, however, we obtain fair agreement with experimental data using a very simple method. The overall mean unsigned error for the 80 mutations is 1.96 kcal/mol; the rms error is 2.73 kcal/mol. The correlation between the computed and measured data is 73%. The rank ordering of the data is characterized by a Spearman rank correlation of 70% [34]; the probability of obtaining this value by chance is less than 0.001 (according to Student's test with a *t*-value of 8.59 and 80 degrees of freedom [34]). Our errors are only slightly higher than those reported by Pei et al., who obtained an rms error of 2.0 kcal/mol for the binding affinities of 50 protein-ligand complexes. Likewise, the accuracy obtained here appears comparable to that achieved by Guérois et al. [40] for their charged mutations, although this subset was not reported separately. Recently, Handel et al. [41] predicted the stabilities of more than 1500 mutants to within 1 kcal/mol. Partly, this improvement might be due to their more sophisticated, all-atom, force field, which increases accuracy but makes their calculations almost an order of magnitude more expensive, compared with the CHARMM19/CASA level (the increased cost being due partly to the explicit treatment of all hydrogens and partly to the need for a more detailed rotamer library). Further, our data set contains a higher percentage of charged mutations, which makes predictions more demanding.

As an additional reference, the performance of the optimized CASA models was compared to that of a GB/SA model, which should provide a more accurate treatment of the electrostatic contribution to the solvation free energy [42]. Only the 55 mutations for systems (i-v) were studied. Two variants of the GB model were tested: (i) GB-HCT [43] in combination with the Amber, all-atom force field [44] and (ii) GB-ACE [45,46] with the CHARMM19 force field [47]. For the GB-HCT variant, the overall quality of the results is close to the CASA level, with a slightly higher mean error (1.96 kcal/mol, Table 3). The GB-ACE variant gives notably higher mean errors for all protein-ligand systems. This might be due to the GB-ACE parameterization which is not optimized for this specific application. The GB-HCT parameters were optimized previously for computational sidechain placement and protein mutagenesis [20]. Some differences between GB-HCT and CASA might be due to the different force field treatments, as Amber uses explicit hydrogens on all atoms, while CHARMM19 uses implicit hydrogens for unpolar atoms. The qualitative agreement with the experimental resistance mutations for Abl:imatinib for the two GB variants is comparable to that obtained using the optimized CASA models (Table 4).

### Protein design

In protein design, the amino acid side chains are mutated and sequences are selected to optimize the folding free energy, using a heuristic search algorithm. In our previous protein design study [48], we obtained good results for 16 different globular proteins using the MF solvation parameters. Here, we consider a subset of those proteins, consisting of 8 SH3 domains. These proteins are used to assess the performance of the new, PHIA solvation parameters for protein design. The protein design calculations were carried out using our Proteins@Home distributed computing platform, with the help of volunteers in several countries. Proteins@Home is discribed in more detail elsewhere [49].

For each protein, 450,000 sequences were generated and amino acid identities relative to the native sequence were calculated. Here, we give (consistent with the literature) the full identities of the designed sequences, even though Cys, Gly, and Pro were not allowed to mutate; see Tables 5 and 6. For further analysis, we selected two subsets of the computed sequences: (i) the 40 sequences with the highest identity scores relative to the native protein ("high-scoring sequences"); (ii) the 40 sequences with the best folding free energies ("low energy sequences"). For these two subsets, the mean sequence identities relative to the corresponding native sequence are given in Table 5. We will not discuss the mean identities over all 450,000 computed sequences, because the values lie very close to those obtained for the low energy sequences. For the low energy subset, the average sequence identity obtained with the PHIA parameters is 32.8%, slightly lower than the value obtained earlier with the MF parameters, 35.0% [48]. For the eight proteins, the identities obtained with PHIA range from 26.5% to 45.1%. The relative performance of the MF and PHIA parameters depends on the protein: since Hck and c-Src are better predicted with PHIA, while 1gcqB, Crk, Abl, and especially Csk are better predicted with MF. Considering the subset of high-scoring sequences, the PHIA parameters give results of roughly the same quality as MF: the mean identities for this subset are 44.4% with PHIA, compared to 46.6% with MF.

**Table 5:** Mean identities (%) for the computed sequences

			MF (*ε *= 10)		PHIA (*ε *= 14)	
PDB code	Name	length	low energy	high score	low energy	high scoring
1gcq(B)	Grb2	57	37.2	49.5	36.1	47.0
1gcq(C)	Vav	69	45.6	55.0	45.1	52.4
1cka	c-Crk	56	39.8	52.1	35.9	49.9
1shg	alpha-spec.	57	26.9	37.6	26.5	39.5
1abo	Abl kinase	58	37.7	48.5	34.7	45.8
1ad5	Hck kinase	58	22.6	38.4	23.6	36.1
1csk	Csk	56	37.3	48.5	27.0	38.5
1fmk	c-Src	60	32.4	43.5	33.8	45.9
	average		34.9	46.6	32.8	44.4

**Table 6:** Blosum scores for computed and natural sequences

PDB code	natural sequences	computed (all)	low energy	high scoring
1gcqB	98.2	67.8	74.0	100.8
1gcqC	43.2	110.5	121.6	141.2
1cka	99.1	57.8	71.2	104.1
1shg	95.9	35.7	38.5	65.2
1abo	89.0	67.3	71.4	98.3
1ad5	111.3	35.5	36.8	75.0
1csk	87.7	60.9	63.0	90.5
1fmk	122.2	56.2	64.2	92.5

With both the PHIA and the MF parameters, the identity scores obtained for the 8 proteins lie within the range of published average identity scores for redesigned proteins [27,28,41,50,51]. In a protein design study by Jaramillo et al. [50] sequence optimizations for 11 SH3 domains were performed and resulted in an average sequence identity of 23.9%. Our energy-ranked PHIA sequences lie well above this score, with a sequence identity of 32.8% averaged over all 8 proteins. Recently, Saunders et al. [28] used a refined protein design method and reported sequence identities as high as 37% for 42 globular proteins. Considering the full sequence identities of our best-scoring sequences, both our parameter sets give results that lie close to this value. Pokala and Handel [41] used an all-atom force field and a GB/SA solvent model to redesign 8 proteins and achieved somewhat higher sequence identities, between 33.5 and 46.7%. This approach, however, includes a negative design criterion, which constrains the surface amino acid composition of the proteins to be native-like. It also leads to an increase in computational effort of about two orders of magnitude, compared with the CASA solvent model and a united-atom force field such as Charmm19.

**Figure 4:**
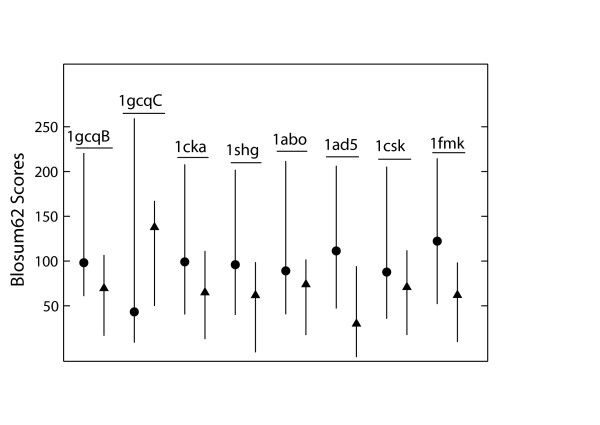
**Comparison of Blosum scores for natural and computed sequences**. For each protein (denoted by the respective PDB code), the vertical lines represent the range of scores within the natural sequence set (left) and the computed sequence set (right). The average score is shown as circles and as triangles for the natural and the computed sequences, respectively.

Next, we addressed the question whether the sequences computed with the new solvation parameters ressemble naturally occuring sequences. As a reference, we created a set of natural sequences in the following way. For each of the 8 proteins, sequences from the SwissProt database with a sequence identity of more than 60% relative to the respective native sequence were retrieved. These sequences were then combined into a large set consisting of 94 sequences. Table 6 compares the average Blosum62 scores obtained for the natural sequence set with those for the complete set of computed sequences and for our two sets of high-ranking sequences (either low energy or high Blosum62 sequences). A weighting scheme was applied to the Blosum scores to account for the variability of each amino acid within the natural sequences. This scheme gives a greater weight to amino acids that are conserved within the natural set. For a frequency of an amino acid of more than 80% at a certain position within the natural sequence set, a weight of 1 was assigned; for a frequency between 50 and 80%, a weight of 0.75 was assigned; for a frequency of less than 50%, a weight of 0.5 was assigned. The Blosum62 scores show that the computed sequences cover a considerable part of the natural sequence set. Figure 4 shows that the computed scores overlap with the range of natural sequences. For most proteins, the weighted average Blosum score lies below the correspinding score for the natural sequence set, but is within the range of the natural sequences. In one case (1gcqC), the weighted average Blosum score even exceeds the value of the natural set. This indicates that the computed sequences do indeed show native-like characteristics and behave like distant homologues of the native sequences.

## Discussion

Simple, efficient, solvent models are of great importance in protein modelling and structural bioinformatics. Here, we have continued to explore the performance of the CASA solvent model, in two directions. First, we considered a variant of increased complexity, where aromatic atoms are treated as a separate group. Our previous approach [20] did not distinguish between unpolar and aromatic atoms. Indeed, the solvation properties of aromatic groups are rather different from other nonpolar groups found in proteins. For this variant, we reparameterized the model completely, leading to the PHIA parameter set. Second, we applied the CASA model to a wider set of applications than previously. We considered protein stability changes associated with point mutations, including all amino acid types (in contrast to our previous work [20]). We also considered protein:ligand binding, an especially important application. Finally, we performed complete protein redesign with the new parameters.

For the new, PHIA parameterization, four different atom types were considered for the atomic surface coefficients: unpolar, aromatic, polar and ionized atoms. The solvation parameters were fitted to a set of 140 experimental stability changes for protein and peptide mutations. Protein stabilities were calculated using an unfolded reference state modelled by a collection of tripeptide structures. These reference structures were taken from a large library of structural fragments from six different proteins. Starting from the earlier, MF solvation parameters [6,20], an iterative procedure was employed, which optimizes, at each cycle, both the solvation parameters and the model for the unfolded reference state of a protein. Atomic parameters were chosen that gave a minimal deviation from the experimental data and also represent the expected relative hydrophobicities of the atom groups. The selected parameter set gives a mean unsigned error of 2.28 kcal/mol for the 140 stability mutations, compared to 3.56 kcal/mol with the MF parameters. Cross-validation tests gave similar parameter values and similar error levels.

For the binding calculations, over 50 experimental mutations in 5 different protein-ligand systems were used, including both small molecule ligands and protein-protein complexes. The calculated differences in binding free energy are in reasonable agreement with the experimental data, with both CASA variants. The mean unsigned error is 1.76 kcal/mol with the PHIA parameters and 1.47 kcal/mol with the MF parameters. It was also shown that the optimized CASA models are not inferior to methods such as GB-ACE/SA or GB-HCT/SA, which treat the electrostatic contribution to solvation more accurately. Two additional protein-protein complexes (BPTI:trypsin, BPTI:chymotrypsin) required a slightly modified protocol to give comparable error levels.

Protein design was carried out for eight SH3 domain proteins and the performance of the PHIA parameters was compared with that of the MF parameters. On average, slightly lower sequence identities with the native sequence were achieved using the new, PHIA parameters. Differences, however, are small, and the performance depends on the particular protein. On the whole, both parameter sets give sequences of comparable quality, which are competitive with recently published results for designed proteins. Further, the computed sequences were found to have the character of naturally occuring, distant homologues of the native sequences.

## Conclusion

Overall, the CASA model performs well for a wide variety of applications. The PHIA parameters, specifically optimized here for protein stability, give a distinct improvement over the earlier, MF parameters for this application. For ligand binding and protein design, the specific treatment of aromatic groups in the PHIA parameterization did not lead to an improvement in performance. Rather, the two parameter sets perform well for both applications, with the exact relative performance dependening on the particular system. Both variants provide an efficient tool for the computational engineering of ligands and proteins.

## Methods

### Effective energy function

The effective free energy function we used in our calculations takes the following form:

(1) *E *= *E*_*bonds *_+ *E*_*Angl *_+ *E*_*Dihe *_+ *E*_*impr *_+ *E*_*vdW *_+ *E*_*Coul *_+ *E*_*solv*_

The first six terms represent the protein internal energy and are taken from the CHARMM19 empirical energy function [47]: a covalent bond energy term, a bond angle energy term, a torsion energy term, an improper dihedral energy term which maintains the chirality or planarity of certain atom centres, a Van der Waals energy term and a Coulomb electrostatic energy term. The last term, *E*_*solv*_, models the effect of the solvent, and represent either a CASA term or a GB term in this study. When using the GB variant HCT for the solvent term, force field parameters for the energy function were taken from Amber [44] (see below).

### Coulomb/Accessible Surface Area (CASA) model

This implicit solvent model uses a screened Coulomb energy term and a solvent accessible surface energy term [3]. The former describes the dielectric screening of solvent-solute interactions. It reduces interactions between solute atoms by a constant factor *ε *to account for the shielding by the high dielectric solvent. The latter term represents local solute-solvent interactions, such as van der Waals energy and cavity energy (creating a cavity against the solvent pressure and reorganization of solvent molecules around the solute), that are assumed to be proportional to the solvent accessible surface area of the solute atoms. The equation for the solvation free energy takes the following form:

(2) $$E_{solv}=E_{screen}+E_{surf}=\left(\frac{1}{\varepsilon }-1\right)E_{coul}+\alpha \sum_{i}\sigma_{i}A_{i}.$$

where *A*_*i *_is the exposed solvent accessible surface area of atom i, and the summation is over all atoms in the solute; *σ*_*i *_(measured in kcal/mol/Å^2^) is a parameter that depends on the nature of atom i and reflects each atom's preference to be exposed or hidden from solvent; *α *is an overall weight applied to the surface energy term. Surface areas were computed by the Lee and Richards algorithm [52], implemented in the XPLOR program [53], using a 1.5 Å probe radius. The solute atoms were divided into 4 groups with characteristic surface coefficients *σ*_*i*_: unpolar, aromatic, polar and ionic. Hydrogen atoms were assigned a surface coefficient of 0. The weight *α *was not optimized during the parameter scans (fixed to 1) but was adjusted in subsequent applications. For the protein design calculations (see below), a value of 1 was used. For the ligand-binding calculations (below), a value of 0.5 worked best. The dielectric constant was optimized in the parameter scans, with a value of 24 working well for the stability mutations. Values of 16 and 14 were used, respectively, for the ligand-binding and protein design calculations.

### Generalized Born/Surface Area (GB/SA) model

A more sophisticated description of electrostatic interactions in a heterogeneous dielectric medium is provided by the Poisson-Boltzmann (PB) equation [13-15]. Given a spatial charge distribution in an environment with one or more dielectric constants, the electrostatic potential can be calculated. The cost involved in solving the PB equation numerically, however, limits its use. A more efficient alternative is the Generalized Born (GB) model [11,12], which is based on PB theory but replaces the solution to the electrostatic potential by an approximate calculation of the solvent-induced reaction field energy. In the GB/SA method, the electrostatic contribution to the solvation free energy is described by a GB term, while the non-polar contribution is modelled as proportional to the solvent accessible surface area of the solute. The two contributions are balanced by a factor *σ *applied to the surface area term:

(3) Esolv=EGB+σ∑iAi

where *E*_*GB *_is the GB term consisting of a self-energy term and an interaction term as described elsewhere [14,20]; *A*_*i *_is the exposed solvent accessible surface area of atom i, and the summation is over all atoms in the solute. In effect, a single surface coefficient *σ *is used for all atom types.

### Calculation of stability changes

In general, introducing a mutation changes the stability of a protein. Here, differences between the stability of a mutant protein and the native protein were calculated. The stability of each protein is computed as the difference in free energy between the folded state and an unfolded reference state. The free energy change upon mutating a protein is thus:

(4) $$\Delta \Delta G=(G_{mut}-G_{mut}^{\text{ref}})-(G_{nat}-G_{nat}^{\text{ref}})$$

where *G*_mut_, *G*_nat _are the free energies of the mutant and native protein, respectively, and $G_{\text{mut}}^{\text{ref}}$, $G_{\text{nat}}^{\text{ref}}$ are the free energies of the unfolded reference state for the mutant and native protein, respectively. The free energy of each state is evaluated using the effective energy function given in equation (1) with the solvent contribution being represented by a CASA term. The nonbonded interactions were cut off at a distance of 10 Å between atoms using a shifting and a switching function for electrostatic and van der Waals interactions, respectively.

The native structures for the folded state were taken from the Protein Data Bank (PDB) [54] with the structure codes 2LZM, 2RN2 and 1STN, respectively. The side chains were slightly minimized prior to any energy evaluation. For the peptide mutations, experimental structures are not available, and models were built using the SwissPDB viewer [55], which constructs an ideal *α *helix from a given sequence. After side chain minimization of the helix models, the structures were treated identically to the protein structures.

The corresponding mutant protein and peptide structures were created by replacing the side chain at the mutated position with the mutant side chain while maintaining all other atom coordinates. The coordinates for the mutant side chain were taken from the Tuffery rotamer library [56]. For each rotamer, the side chain was minimized (30 steps of Powell minimization), with the backbone fixed, and the energy of the mutant protein was evaluated. The mutant side chain rotamer giving the lowest energy for the mutant protein was retained. For the native structures, the side chain conformation at the position to be mutated was kept as in the crystal structure and only subjected to a short minimization (30 steps). In some cases, unfavourable van der Waals contacts in the proteins occured upon mutation. These data were considered as outliers and were not used for parameter adjustment (see below).

The experimental stability changes for the protein mutations were taken from the ProTherm database [57]. For the peptide stability changes, experimental helix propensity scales were used. The sequences of the various peptide systems are given below, with the mutated position denoted by X:

pepT1: SSDVSTAQXAAYKLHED [58],

KEAKE: YEAAAKEAXAKEAAAKA [59],

K2AE2: YSEEEEKAKKAXAEEAEKKKK [60],

VAR: KETAAAKFERQHMDS [61],

PAD: YKAAAAKAAXAKAAAAK [62],

KAL: YSEEEEKKKKXEEEEKKKK [63],

SH1: AETAAAKFERQHM [64],

SH2: KETAAAKFERAHA [64]

### Unfolded state

In the unfolded state of a protein, it is assumed that amino acid side chains do not interact with each other, but only with nearby backbone groups and with solvent. This situation can be modelled by a collection of n tripeptide structures with the sequence Ala-X-Ala; n is the number of amino acids in the protein. For each amino acid type X, a number of possible structures with different backbone and side chain conformations were considered. These structures were extracted from various positions in the X-ray structures of 6 different proteins taken from the PDB [54]: lysozyme (2LZM), bovine pancreatic trypsin inhibitor (4PTI), staphylococcal nuclease (1STN), *α*-toxin (1PTX), ribonuclease A (2RN2) and cyclophilin (2CPL). In each tripeptide structure, the side chain X was slightly minimized with respect to itself and the backbone of the whole tripeptide. To choose the optimal tripeptide structure for each amino acid type, the interaction between the respective side chain and the tripeptide backbone served as a criterium. Thus, for each amino acid type X, the tripeptide structure giving the lowest interaction energy was taken to represent the preferred structure for X in the unfolded state. The total free energy of the unfolded state is obtained by summing the contributions, E_*X*_, of the n individual amino acids of the protein. When comparing the folding free energies of two sequences, only sidechain – sidechain and sidechain – backbone interactions are taken into account. Interactions between different portions of the backbone cancel, both in the folded and the unfolded state, so that no important interactions are missed through the tripeptide unfolded model.

### Iterative optimization

Since the choice of the reference structure for each amino acid depends on the set of parameters used in the CASA model, the solvent parameters and tripeptide structures had to be optimized iteratively (Figure 1). As a starting point for optimization, we took the surface parameters developed by Fraternali and van Gunsteren [6], supplemented by an additional surface coefficient for ionic atoms [20]. In this initial parameter set, aromatic atoms were assigned the same surface coefficient as unpolar atoms.

The atom groups were assigned as follows: (i) unpolar: all alkane carbons, the carbonyl carbons of the protein backbone, and S; (ii) aromatic: Trp, Phe and Tyr aromatic ring carbons and nitrogens; (iii) polar: N/O atoms not belonging to ionized groups, N-C-N group in the His ring; (iv) ionic: guanidinium group of Arg, carboxyl group of Asp/Glu, N-C-N group in the ring for protonated His.

Using this solvation model, a first choice of reference structures was made. Then, stability changes were calculated according to equation (3) using the current set of reference structures. These stability changes were calculated for all combinations of surface parameters within a range of values given below (Table 7). Thus, for each combination of parameters, a set of ΔΔG values was obtained; a mean unsigned error and an rms deviation from the experimental stability changes were determined. The rms deviation was used as a score for the performance of a given surface parameter combination. From the top-scoring parameter sets, a physically meaningful set was chosen and used as a new starting point for the next iterative optimization cycle. This procedure was repeated until no further significant change in the ranking of the parameter combinations occurred.

**Table 7:** Range of solvation parameters (kcal/mol/Å^2^) and dielectric values *ε *scanned during the iterative optimization

atom type	range	interval
unpolar	-0.005 to 0.01	0.005
aromatic	-0.08, -0.06 to 0.01	0.01
polar	-0.12, -0.10 to -0.04	0.01
ionic	-0.20, -0.18 to -0.10	0.01
*ε*	16 to 32	8

### Cross validation

The optimization procedure was tested for bias and overfitting by the following cross-validation procedure. 30 of the 140 mutants were chosen randomly and left out of the optimization. The reference structures were taken from the above, iterative optimization and kept fixed. Parameter scanning was then performed, leading to several good quality parameter sets. The mean errors were then computed for the omitted, or "test" data. This procedure was done twice, with two distinct sets of mutations omitted from the optimization. The parameter sets and error levels from these two runs were similar to each other and to the iterative optimization described above, showing that the optimization is not subject to excessive overfitting or bias.

### Binding affinities

Binding affinities were calculated for 5 different ligand-protein systems taken from the PDB [54] and a number of their mutants: (i) tyrosine kinase Abl in complex with imatinib (1OPJ), (ii) Tyrosyl-tRNA synthetase in complex with tyrosine (4TS1), (iii) Aspartyl-tRNA synthetase in complex with aspartate (1IL2), (iv) Lysozyme in complex with the antibody HyHel-10 (3HFM) and (v) the complex of the glycoprotein CD4 with the gp120 component of the HIV virus (1G9M). As starting structures, the ligand-bound X-ray structures of these 5 proteins were used. The system (iii) was truncated to a 30Å sphere around the ligand; systems (ii), (iv) and (v) were truncated to 20 Å spheres around the ligand, and for system (i) the untruncated structure of chain B was used. The mutant structures were created by replacing the side chain at the relevant position with a rotamer of the mutant side chain from the Tuffery library [56].

A simple protocol was adopted for the energy evaluation: The side chain at the mutated position was subjected to 50 steps of minimization with respect to itself and to all other side chains with the backbone kept fixed. During this minimization, all sidechains were allowed to adjust to the introduced mutation but otherwise inter-sidechain interactions were excluded. The energy of this slightly adjusted protein conformation was taken as the ligand-bound energy. For the ligand-free state, the ligand was removed, the side chains were again minimized and the energy of this conformation was taken. For the mutated sidechain, all rotamers from the library were considered, and the lowest energy for the ligand-bound and ligand-free states, respectively, was retained. In the native structure, the rotamer at the position to be mutated was not varied, as it is assumed that the X-ray structure already represents a low energy conformation.

Three different solvent treatments were employed in this protocol:

(1) The CASA model as described above using the parameters optimized earlier [20], with a weight factor *α *of 0.5 and a dielectric constant *ε *of 16. We obtained good results for sidechain placement and stability changes in our previous work [20] using these values.

(2) The CASA model with the parameters optimized here, with the same weight factor *α *of 0.5 and dielectric constant *ε *of 16. These values of *α *and *ε *were chosen because they gave the best agreement with the experimental binding affinities.

(3) A Generalized Born/Surface Area (GB/SA) model with a weight factor *σ *of -0.05 kcal/mol/Å^2 ^for the surface term and a dielectric constant *ε *of 8.0.

Mutations for which the van der Waals energy contributed more than 10 kcal/mol to the difference in binding energy were considered outliers and were not included in the results. These contributions are probably due to unfavourable contacts that are not resolved by the simple minimization protocol used here.

For two additional systems, a slightly different protocol gave distinctly better results. These were the BPTI:trypsin and BPTI:chymotrypsin complexes [38,39]. Several BPTI mutations at position 15 (within the interface) were considered. Instead of minimizing just the mutated sidechain, as above, we minimized the entire BPTI protein for each choice of rotamer (for 50 steps, as above).

### Protein design

The energy function for protein design corresponds to Equation (1), with the solvation contribution described by a CASA term. The interaction energy between each possible combination of sidechain pairs, or between a sidechain and the backbone, are precomputed and stored in an energy matrix. For a given sidechain pair, this calculation includes all possible combinations of both amino acid types and rotamer values. Once the energy matrix is computed, the amino acid sequence is optimized in a second stage, through cycles of random mutations and steepest-descent minimization. This heuristic procedure was developed and validated by Wernisch et al. [24]. A "heuristic cycle" proceeds as follows. An initial amino acid sequence and set of sidechain rotamers are chosen randomly. These are improved in a stepwise way. At a given amino acid position *i*, the best amino acid type and rotamer are selected, with the rest of the sequence and structure held fixed. The same is done for the following position *i *+ 1, and so on, performing multiple passes over the amino acid sequence until the energy no longer improves (or a given, large number of passes is reached). The final sequence, rotamer set, and energy are output, ending the cycle. For the design calculations below, we performed 450,000 heuristic cycles for each protein. Disulfide-bonded cysteines, glycines and prolines are expected to have a special effect on the protein's folded and unfolded state structures, which may not be accurately captured by our method. Therefore, if these amino acids were present in the native sequence, they were held fixed; all other amino acids were allowed to mutate freely. The calculations were done using our Proteins@Home distributed computing platform. This allows us to use the computers of several thousand volunteers in over 70 countries. Proteins@Home is based on the Berkeley Open Infrastructure for Network Computing, BOINC [65]. The Proteins@Home platform and project will be described in detail elsewhere [49].

The model for the unfolded state of a protein is analogous to that described above, employing a collection of tripeptide structures. However, an additional, empirical correction was added to the unfolded state energies. For each amino acid type, the correction is defined and optimized to provide a realistic overall amino acid composition of the resulting sequences. More details of this procedure are given elsewhere [48]. The precise values of the reference energies are given in Table 8. The dielectric constant was set to *ε *= 14; the weight of the surface term (Eq. 2) was set to *α *= 1. Notice that our earlier design calculations with the MF parameters [48] used *ε *= 10, *α *= 1, and different reference energies.

**Table 8:** Reference energies (kcal/mol) characterizing the unfolded state

	initial	optimized	difference
Ala	-10.009	-11.307	1.30
Asp	-24.223	-19.826	-4.40
Asn	-20.783	-17.180	-3.60
Arg	-22.199	-25.043	2.84
Glu	-24.365	-21.257	-3.11
Gln	-20.707	-17.940	-2.77
His	-21.928	-20.389	-1.54
Ile	-13.904	-12.320	-1.58
Leu	-13.941	-12.600	-1.34
Lys	-18.946	-22.214	3.27
Met	-14.013	-13.922	-0.09
Phe	-21.741	-17.412	-4.33
Ser	-16.656	-13.450	-3.21
Tyr	-23.727	-20.274	-3.45
Thr	-16.252	-12.583	-3.67
Trp	-23.993	-20.983	-3.01
Val	-13.338	-11.481	-1.86

## Authors' contributions

MSAB: wrote software, performed calculations, analyzed data, wrote paper. AL: wrote software, performed calculations, analyzed data. NA: performed calculations, analyzed data. CB: Performed calculations, analyzed data, wrote paper. TS: designed research, wrote software, wrote paper. All the authors read and approved the final manuscript.

## Supplementary Material

Additional file 1 Calculated and experimental binding free energies. Complete details on the binding free energy changes due to point mutations in the various test proteins and peptides. Click here for file
