# Supplementary material for: Testing the Coulomb/Accessible Surface Area solvent model for protein stability, ligand binding, and protein design
Source: BMC Bioinformatics. 2008 Mar 13;9:148. doi: 10.1186/1471-2105-9-148 (PMC2292695; doi:10.1186/1471-2105-9-148)
Supplement: Additional file 1 — Calculated and experimental binding free energies. Complete details on the binding free energy changes due to point mutations in the various test proteins and peptides. [file 1471-2105-9-148-S1.pdf]

Supplementary Material
Testing the Coulomb/Accessible Surface Area solvent model for protein stability, ligand binding, and protein design

Marcel Schmidt am Busch, Anne Lopes, Najette Amara, Christine Bathelt & Thomas Simonson*

Laboratoire de Biochimie (CNRS UMR7654), Department of Biology, Ecole Polytechnique, 91128 Palaiseau, France.

*Email: thomas.simonson@polytechnique.fr

Table SM1 gives the experimental and computed binding free energy changes for the six proteins used as test systems: aspartyl-tRNA synthetase (AspRS), tyrosyl-tRNA synthetase (TyrRS), the lysozyme antibody complex (Lyso), the CD4:gp120 complex (CD4), the BPTI:trypsin complex (trypsin), and the BPTI:chymotrypsin complex (chymo).

Table SM2 gives the stability changes for the helical peptides used as test systems.

Table SM1 - Calculated and experimental differences in binding affinity $\Delta\Delta G$ in kcal/mol for small molecule ligands

			CASA		GB-ACE		GB-HCT	
Protein	mutation	$\Delta\Delta G_{exp}$	$\Delta\Delta G_{calc}$	|error|	$\Delta\Delta G_{calc}$	|error|	$\Delta\Delta G_{calc}$	|error|
AspRS	Q195A	1.59	2.06	0.47	-2.57	4.16	2.54	0.95
	Q195E	1.69	0.85	0.84	-8.8	10.48	-1.2	2.89
	Q195N	2.27	-0.97	3.24	-4.58	6.85	1.88	0.39
	K198L	1.0	4.97	3.97	2.35	1.35	1.91	0.91
	Q199A	1.68	0.05	1.63	4.12	2.44	1.83	0.15
	Q199E	0.7	-0.15	0.85	2.69	1.99	0.15	0.55
	Q199N	1.74	0.07	1.67	2.17	0.43	-0.12	1.86
	E235D	1.06	-0.15	1.21	1.02	0.04	-	-
	D233E	1.68	-	-	-	-	-0.16	1.84
	R489H	1.35	4.17	2.82	4.24	2.89	-0.36	1.71
TyrRS	Y34F	1.4	2.74	1.34	1.24	0.16	0.52	0.88
	C35S	-0.24	0.07	0.31	-0.13	0.11	0.09	0.33
	D38A	2.41	3.6	1.19	2.8	0.39	1.81	0.6
	T51A	0.0	0.23	0.23	0.15	0.15	-0.29	0.29
	D78A	2.61	4.55	1.94	3.45	0.84	4.42	1.81
	N123A	0.93	0.82	0.11	0.63	0.3	0.91	0.02
	N123D	3.33	0.8	2.53	-0.89	4.22	-1.58	4.91
	Y169F	2.79	3.21	0.42	-1.03	3.82	-1.44	4.23
	Q173A	2.31	2.4	0.09	3.07	0.76	3.37	1.06
	K230A	0.38	0.02	0.36	-0.09	0.47	-0.32	0.38
	F231L	0.0	0.01	0.01	-0.07	0.07	0.39	0.39
	G232A	0.41	0.02	0.39	-0.09	0.5	0.19	0.22
	K233A	-0.08	0.01	0.09	-0.08	0.0	-0.31	0.23
	T234A	-0.17	0.01	0.18	-0.1	0.07	0.18	0.35
	T234S	0.05	0.01	0.04	-0.1	0.15	-0.92	0.97

Table SM1, continued - Calculated and experimental differences in binding affinity

			CASA		GB-ACE		GB-HCT	
Protein	mutation	$\Delta\Delta G_{exp}$	$\Delta\Delta G_{calc}$	$\lvert$error$\rvert$	$\Delta\Delta G_{calc}$	$\lvert$error$\rvert$	$\Delta\Delta G_{calc}$	$\lvert$error$\rvert$
Lyso	R21E	2.5	4.39	1.89	1.4	1.1	6.62	4.12
	R21K	1.9	5.51	3.61	5.1	3.2	6.44	4.54
	W62Y	-0.1	-0.9	0.8	-0.89	0.79	0.64	0.74
	W63Y	1.7	1.36	0.34	1.88	0.18	4.71	3.01
	K96M	6.7	2.0	4.7	-0.63	7.33	8.54	1.84
	K97D	6.7	5.13	1.57	-0.51	7.21	-	-
	K97G	6.4	7.71	1.31	5.52	0.88	9.1	2.7
	K97M	0.9	5.06	4.16	3.17	2.27	-	-
	D101G	0.5	6.24	5.74	4.65	4.15	8.12	7.62
CD4	S19Y	0.17	0.02	0.15	-0.9	1.07	0.99	0.82
	H27A	0.34	-0.29	0.64	1.68	1.34	2.13	1.79
	G38A	1.4	0.02	1.38	-0.65	2.05	2.16	0.76
	Q40A	-0.35	3.03	3.38	-1.58	1.23	4.57	4.92
	F43L	0.61	3.12	2.51	-	-	-	-
	F43W	1.71	5.74	4.03	-	-	-	-
	F43Y	2.13	-1.78	3.91	-	-	-	-
	T45A	-0.24	-0.26	0.02	-4.33	4.09	3.34	3.58
	K46A	1.82	1.70	0.12	-1.29	3.11	12.12	10.3
	P48Q	0.96	-1.71	2.67	-1.14	2.1	3.62	2.66
	S49V	1.59	-0.06	1.65	4.49	2.9	1.01	0.58
	N52A	-0.07	1.31	1.38	-0.43	0.36	3.16	3.23
	A55V	0.58	-0.03	0.61	-7.89	8.47	0.92	0.34
	D56A	0.34	-0.21	0.55	4.04	3.7	-2.97	3.31
	R59A	1.29	7.96	6.67	0.95	0.34	-	-
	R59K	-0.24	1.91	2.15	2.51	2.75	-	-
	W62Y	3.06	0.07	2.99	4.53	1.47	3.96	0.9
	E77Q	0.22	0.02	0.20	0.97	0.75	-2.35	2.57
	T81A	0.3	0.02	0.28	-1.81	2.11	1.57	1.27
	E85A	0.22	0.14	0.08	3.64	3.42	-1.79	2.01
	E85Q	0.3	0.00	0.30	2.67	2.37	-2.3	2.6
	Q89L	0.17	2.13	1.96	0.95	0.78	1.49	1.32

Table SM1, continued - Calculated and experimental differences in binding affinity

			CASA	
Protein	mutation	$\Delta\Delta G_{exp}$	$\Delta\Delta G_{calc}$	|error|
Chymo	K15A	2.19	5.19	3.00
	K15S	3.39	4.64	1.25
	K15V	2.13	0.62	1.51
	K15T	2.08	2.03	0.05
	K15L	-1.58	-3.01	1.43
	K15I	2.93	12.14	9.21
	K15M	-1.41	-2.73	1.32
	K15N	1.32	0.03	1.29
	K15Q	0.31	-3.07	3.38
	K15H	0.10	-7.08	7.18
	K15P	-1.96	-4.14	2.18
	K15Y	-2.61	-6.07	3.46
	K15W	-2.43	-3.76	1.33
Trypsin	K15A	10.69	12.20	1.51
	K15S	7.77	10.82	3.05
	K15V	11.82	11.04	0.78
	K15T	10.65	9.70	0.95
	K15L	8.91	4.78	4.13
	K15I	11.23	10.33	0.90
	K15M	7.74	3.60	4.14
	K15N	8.07	5.75	2.32
	K15Q	9.42	5.10	4.32
	K15H	8.83	1.32	7.51
	K15P	7.07	7.34	0.27
	K15Y	6.89	9.18	2.29

Table SM2 - Calculated and experimental stability changes for individual mutations

mutation	$\Delta\Delta G_{mut}$	exp	|error|
KEA_A9D	0.9	1.23	0.2
KEA_A9E	1.13	0.93	0.74
KEA_A9F	1.17	0.45	0.72
KEA_A9H	0.15	1.17	1.02
KEA_A9I	2.20	0.78	1.42
KEA_A9K	1.12	0.20	0.92
KEA_A9L	0.46	0.31	0.15
KEA_A9M	0.30	0.45	0.15
KEA_A9N	-0.60	1.12	1.72
KEA_A9Q	-0.74	0.62	1.36
KEA_A9R	-1.04	-0.01	1.03
KEA_A9S	2.64	1.01	1.63
KEA_A9T	2.04	1.25	0.79
KEA_A9V	2.24	1.25	0.99
KEA_A9W	-1.43	0.62	2.05
KEA_A9Y	-1.11	0.78	1.89
K2A_A12D	-2.07	0.78	2.85
K2A_A12E	-1.88	0.21	2.09
K2A_A12F	-1.68	1.11	2.79
K2A_A12H	-2.74	0.54	3.28
K2A_A12L	-2.40	0.36	2.76
K2A_A12M	-2.60	0.53	3.13
K2A_A12N	-3.49	0.85	4.34
K2A_A12Q	-3.66	0.80	4.46
K2A_A12R	-3.97	0.41	4.38
K2A_A12S	-0.22	0.76	0.98
K2A_A12T	-0.87	0.89	1.76
K2A_A12V	-0.63	0.73	1.36
K2A_A12W	-4.13	1.11	5.24
K2A_A12Y	-4.11	1.95	6.06
K2A_A12I	-0.64	0.38	1.02
K2A_A12K	-1.95	0.59	2.54

Table SM2, continued - Calculated and experimental stability changes for individual mutations

mutation	$\Delta\Delta G_{mut}$	exp	|error|
SH1_A5H	4.18	0.84	3.34
SH2_E9L	-0.26	1.10	0.49
PT1_A9D	-2.37	0.68	3.05
PT1_A9E	-0.85	0.31	1.16
PT1_A9F	-1.83	0.60	2.43
PT1_A9H	-2.66	0.67	3.33
PT1_A9L	-1.98	0.22	2.20
PT1_A9M	-2.30	0.15	2.45
PT1_A9N	-3.68	0.62	4.30
PT1_A9Q	-3.81	0.30	4.11
PT1_A9R	-3.52	0.47	3.99
PT1_A9S	-0.27	0.47	0.74
PT1_A9T	0.21	0.65	0.44
PT1_A9V	0.66	0.64	0.02
PT1_A9W	-3.91	0.08	3.99
PT1_A9Y	-4.06	0.38	4.44
PT1_A9I	0.86	0.34	0.52
PT1_A9K	-0.95	0.38	1.33
PAD_A10V	-0.57	0.83	1.40
KAL_A11L	-2.72	0.19	2.91
KAL_A11M	-2.90	0.24	3.14
KAL_A11Q	-3.97	0.33	4.30
KAL_A11I	-1.10	0.42	1.52
KAL_A11V	-0.91	0.47	1.38
KAL_A11S	-0.10	0.51	0.61
KAL_A11T	-0.90	0.56	1.46
KAL_A11N	-3.37	0.60	3.97
KAL_A11W	-4.78	0.28	5.06
KAL_A11F	-2.23	0.32	2.55
KAL_A11Y	-4.55	0.53	5.08
KAL_A11H	-2.80	0.63	3.43

Table SM2, continued - Calculated and experimental stability changes for individual mutations

mutation	$\Delta\Delta G_{mut}$	exp	|error|
VAR_M13V	-0.48	0.00	0.48
VAR_M13A	1.68	0.00	1.68
VAR_M13I	-0.50	0.00	0.50
VAR_M13L	-0.18	0.00	0.18
STN_D19A	-0.50	0.10	0.60
STN_D21A	-6.33	-0.70	5.63
STN_D40A	0.88	-0.20	1.08
STN_D95A	4.08	3.30	0.78
STN_E101A	1.50	1.90	0.40
STN_E10A	-0.15	1.30	1.45
STN_E122A	-0.44	0.40	0.84
STN_E129A	3.36	2.40	0.96
STN_E135A	-0.00	0.70	0.70
STN_E52A	0.57	0.10	0.47
STN_E57A	1.94	0.20	1.74
STN_E67A	1.60	1.00	0.60
STN_E73A	3.37	1.20	2.17
STN_E75A	2.93	2.20	0.73
STN_K110A	0.67	1.30	0.63
STN_K116A	-6.30	-0.70	5.60
STN_K127A	0.52	-0.20	0.72
STN_K133A	-0.45	1.40	1.85
STN_K136A	1.85	0.90	0.95
STN_K16A	-2.00	0.20	2.20
STN_K24A	1.39	0.20	1.19
STN_K48A	1.00	-0.10	1.10
STN_K49A	-2.53	0.30	2.83
STN_K53A	-0.08	0.20	0.28
STN_K63A	-4.55	0.50	5.05
STN_K64A	-3.29	-0.10	3.19
STN_K6A	-0.44	-0.30	0.14
STN_K70A	-1.64	0.10	1.74

Table SM2, continued - Calculated and experimental stability changes for individual mutations

mutation	$\Delta\Delta G_{mut}$	exp	|error|
STN_K78A	-3.95	0.60	4.55
STN_K84A	-0.89	-0.20	0.69
STN_K9A	-0.61	1.40	2.01
STN_N118D	4.33	2.40	1.93
STN_R105A	0.97	1.40	0.43
STN_R126A	2.03	1.70	0.33
STN_R81A	2.57	1.10	1.47
STN_K28A	-3.58	0.70	4.28
STN_K97A	-5.83	0.10	5.93
STN_K71A	2.13	0.40	1.73
STN_K134A	-5.16	-0.10	5.06
RN2_K91R	-6.21	0.60	6.81
RN2_D94E	-0.44	0.40	0.84
RN2_K95A	-0.79	-0.10	0.69
RN2_K95N	-4.02	-0.90	3.12
RN2_A52E	6.71	1.50	5.21
RN2_A52D	1.83	2.30	0.47
RN2_D10N	-6.78	0.70	7.48
RN2_D10A	-5.95	-2.40	3.55
RN2_D10E	1.18	-1.10	2.28
RN2_D10S	-4.75	-0.70	4.05
RN2_E48Q	-3.46	-0.30	3.16
RN2_E48A	2.80	0.10	2.70
RN2_E48D	-1.73	-0.20	1.53
RN2_D70N	-3.38	0.30	3.68

Table SM2, continued - Calculated and experimental stability changes for individual mutations

mutation	$\Delta\Delta G_{mut}$	exp	|error|
RN2_D70A	-2.23	0.10	2.33
RN2_D134N	-8.15	0.10	8.25
RN2_D134A	-3.78	-0.80	2.98
RN2_D134V	-3.64	-0.31	3.33
RN2_H62R	-9.54	0.03	9.57
RN2_E135K	0.25	0.22	0.03
LZM_D47A	5.13	0.95	4.18
LZM_D92N	4.18	1.40	2.78
LZM_E45A	-3.69	-0.10	3.59
LZM_K43A	1.08	1.03	0.05
LZM_K48A	-5.71	0.56	6.27
LZM_N144D	-0.90	-0.50	0.40
LZM_Q123E	-0.04	-0.40	0.36
LZM_D20A	2.64	0.30	2.34
LZM_E11A	-1.46	-1.10	0.36
LZM_E128A	-1.07	0.16	1.23
LZM_N116D	0.18	-0.60	0.78
LZM_N40D	-0.62	-0.44	0.18
LZM_Q105E	-0.33	1.10	1.43
LZM_R119A	-0.85	0.18	1.03
